# 15 Years of Intermittent Therapy With Hydroxychloroquine Without Any Loss of Efficacy in Reticular Erythematous Mucinosis

**DOI:** 10.1155/crdm/8309221

**Published:** 2025-05-22

**Authors:** Valeria Brazzelli, Nicolò Di Giuli, Alice Bonelli, Martina Volonté, Eugenio Isoletta

**Affiliations:** ^1^Università Degli Studi di Pavia Dipartimento di Scienze Clinico Chirurgiche Diagnostiche e Pediatriche, Pavia, Italy; ^2^Fondazione IRCCS Policlinico San Matteo, Clinica Dermatologica Pavia, Pavia, Italy

**Keywords:** long-term care, photodermatitis, photosensitivity, reticular erythematous mucinosis

## Abstract

Reticular erythematous mucinosis (REM) is a rare dermatological condition characterized by erythematous, reticulated patches and plaques with a slightly infiltrated appearance. REM is classified among cutaneous mucinoses, which are characterized by the accumulation of mucin in the subcutaneous tissues, leading to the formation of characteristic reticulated patches on the skin. The pathogenesis is still debated, but the association with sun exposure seems to play an important role. We present the case of a 54-year-old individual with a history of recurrent erythema on the chest and back. The patient came to our attention in 2007 due to a worsening of symptoms after sun exposure. On clinical examination, the patient presented with pruritic, erythematous patches with a reticulated appearance. A biopsy was performed, which showed the presence of a moderate lymphocytic infiltrate in the dermis, with a perivascular and periappendageal distribution, consisting mainly of T lymphocytes, and deposits of mucin in the superficial dermis, dissecting the collagen bundles. A diagnosis of REM was made, and the patient began treatment with hydroxychloroquine 200 mg per day, with rapid improvement of symptoms. Considering the resolution of symptoms, the therapy was discontinued after 3 months. The patient remained in remission until the following summer when the condition recurred, requiring a new cycle of hydroxychloroquine therapy. The patient has experienced recurrences over the past 15 years during the summer, which responded to hydroxychloroquine therapy, consistently achieving rapid symptom resolution without ever experiencing loss of efficacy or side effects.

## 1. Introduction

Reticular erythematous mucinosis (REM) is an uncommon, enduring condition that manifests as an erythematous, papular, or plaque-like eruption generally along the central region of back and chest. Affected skin is frequently asymptomatic, but occasionally it can be associated to itch or pain [1].

Since the exact aetiology remains unclear, therapy is often challenging [2]. We present a case of REM patient followed for 15 years. Every year the patient suffered from a recurrence of the disease in summer months. Over the course of 15 years, for each of these recurrences, he was treated with hydroxychloroquine, which maintained its effectiveness, leading to complete resolution of symptoms.

## 2. Case Report

**Figure 1:**
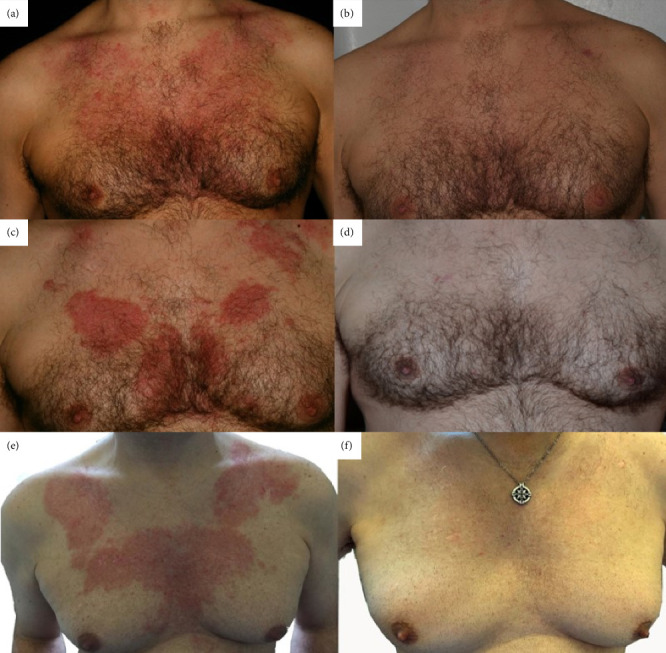
Reticulated erythema on patient's chest: in 2007, before (a) and after (b) treatment with hydroxychloroquine; in 2011, before (c) and after (d) treatment with hydroxychloroquine; in 2022 before (e) and after (f) treatment with hydroxychloroquine.

**Figure 2:**
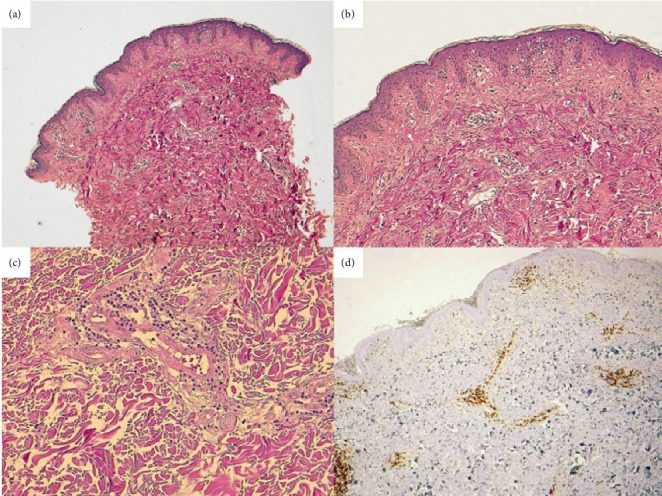
(a) Skin biopsy shows discrete dermal lymphocytic infiltration with a perivascular and peri-adnexal distribution, mainly consisting of peripheral T lymphocytes without cytological atypia. In the superficial dermis, presence of mucin deposits dissecting collagen bundles. (b) Epidermis is almost uninvolved, except for a small portion of vacuolar degeneration. (c) Perivascular lymphocytic infiltration is evident in mid-dermis; (d) CD3 stain, shows diffuse CD3+ lymphocytic infiltration in superficial and mid dermis.

A 54-year-old man presented for the first time to our Department in 2008, with a history of reticulated erythema on his chest and upper back, associated to occasional pruritus and sunlight exacerbation. During examination, the patient showed itchy erythematous patches characterized by a reticular appearance (Figure 1(a)). He reported that previous treatment with topical and systemic steroid therapy and ultraviolet (UV) protection had not provided any improvement. Laboratory tests, including complete blood count, total protein, liver, kidney and thyroid function, were negative or within normal range. Autoantibodies and immunological markers were negative. In the light of these findings, a skin biopsy was performed. Histological examination showed (Figure 2) a discrete dermal lymphocytic infiltration with perivascular and periadnexal distribution, mainly consisting of peripheral T lymphocytes with mild predominance of CD4+ and no cytological atypia. In the superficial dermis, mucin dissecting collagen bundles was present. The direct immunofluorescence (DIF) studies were negative. Correlation between the clinical presentation, histological findings and DIF suggested a diagnosis of REM. The patient started a therapy with hydroxychloroquine 200 mg daily, with complete remission of symptomatology in 3 months (Figure 1(b)). Therefore, the therapy was interrupted. He maintained complete remission until the following summer, when a recurrence occurred. A new cycle of hydroxychloroquine was administered, leading again to complete recovery in 3 months. Since then, the patient presented a relapse of the skin disease every summer for 15 years (Figures 1(c), 1(d), 1(e), and 1(f)), with prompt resolution of symptoms with hydroxychloroquine, without any loss of efficacy of the drug nor adverse effects. Regular eye checks and blood chemistry tests were always performed. When the patient tried to manage the disease only with photoprotection, waiting for spontaneous remission, the attempt was unsuccessful. The extent of the manifestations forced again the use of hydroxychloroquine during summer, which led to complete remission after 3 months.

## 3. Discussion

REM is considered an idiopathic, primary form of mucinosis [3]. Mucinoses refer to a group of rare dermatologic conditions where an excessive production of mucin represents the main pathological process. This group includes disorders such as generalized or localized lichen myxedematous, scleredema, alopecia mucinosa and REM [4]. Mucin can also accumulate in the skin as a secondary process in conditions like lupus erythematosus, dermatomyositis, Degos disease, granuloma annulare, following treatments such as psoralen plus UV A (PUVA) or retinoids. REM is characterized by histological and clinical resemblance to lupus erythematosus tumidus (LET), dermatomyositis, scleredema and lichen myxedematosus. Prompt recognition and correct diagnosis of REM is desirable, given that REM has usually a more favourable outcome. Recently, Palmisano et al. described the in-vivo appearance of mucinoses using LC-OCT, showing its close resemblance to histology, suggesting it as a promising tool for early diagnosis in equivocal lesions [5].

In literature, it is discussed whether REM represents an isolate disease or a possible manifestation of lupus erythematosus [3, 6]. Based on common pathogenetic aspects, clinical symptoms and histological findings, some researchers suggest that REM may be part of the spectrum of lupus erythematosus-like diseases [7]. Among the different manifestation of LE, it appears that REM shares most similarities with LET. Histologically, both diseases are characterized by perivascular dermal infiltrates of lymphocytes, mostly CD4+ positive, and by an increased level of dermal mucin. However, REM often displays more scattered and superficial lymphocytes with less frequent immunoglobulin and complement depositions along the dermo-epidermal junction and a more superficial distribution of mucin compared to LET. LET shows frequently DIF positivity, on the contrary REM is rarely DIF positive [7]. Moreover, both conditions usually lack serologic abnormalities, respond well to antimalarial agents [8].

Despite their common features, REM presents distinct clinical characteristics compared to LET, including a reticular pattern that typically affects the midlines of the chest and back, higher incidence in middle-aged women and possible association with malignancies and thyroid dysfunction [6]. In REM, the relationship of the disease with photosensitivity is controversial, whereas its correlation with the onset of LET is suggested. In our patient, the peculiar clinical presentation, the course of the disease, DIF and histopathological findings suggested the diagnosis of REM.

A deeper understanding of the pathogenesis of this disorder could help in order to have more effective and long-lasting therapeutic approaches, given that, due to the rarity of the disease, there are no established guidelines for the therapy. Antimalarials are considered the treatment of choice [1]. In nonresponsive cases or when contraindicated, therapy with topical and systemic corticosteroids, topical tacrolimus, oral antihistamines, tetracycline, cyclosporine, and UVB irradiation have been used with variable results [1]. In our patient hydroxychloroquine has showed a complete remission in 3 months, every summer. To conclude, despite limited to a single case, our 15-years' experience with prolonged use of this molecule to treat REM supports the knowledge of its safety and prolonged efficacy even for this specific, rare condition.

## Data Availability

The data that support the findings of this study are available on request from the corresponding author. The data are not publicly available due to privacy or ethical restrictions.
