# Correction to “15 Years of Intermittent Therapy With Hydroxychloroquine Without Any Loss of Efficacy in Reticular Erythematous Mucinosis”

**DOI:** 10.1155/crdm/9802656

**Published:** 2025-11-19

Brazzelli Valeria, Di Giuli Nicolò, Bonelli Alice, Volonté Martina, and Isoletta Eugenio, “15 Years of Intermittent Therapy With Hydroxychloroquine Without Any Loss of Efficacy in Reticular Erythematous Mucinosis,” *Case Reports in Dermatological Medicine* (2025): 20258309221, https://doi.org/10.1155/crdm/8309221.

To comply with funding requirements, the authorship order and affiliations have been rearranged. The correct order is displayed below:

Valeria Brazzelli^1,2^, Nicolò Di Giuli^2^, Alice Bonelli^2^, Martina Volonté^2^, and Eugenio Isoletta^2^

^1^Università Degli Studi di Pavia Dipartimento di Scienze Clinico Chirurgiche Diagnostiche e Pediatriche, Pavia, Italy

^2^Fondazione IRCCS Policlinico San Matteo, Clinica Dermatologica Pavia, Pavia, Italy

A funding statement for this article was missing. The following funding statement has been added to the article: Open access publishing has been facilitated by Universita degli Studi di Pavia, as part of the Wiley-CRUI-CARE agreement.

The online version of this article has been corrected accordingly.

We apologize for these errors.

